# Supplementary material for: Mechanisms governing the pioneering and redistribution capabilities of the non-classical pioneer PU.1
Source: Nat Commun. 2020 Jan 21;11:402. doi: 10.1038/s41467-019-13960-2 (PMC6972792; doi:10.1038/s41467-019-13960-2)
Supplement: Supplementary file 7 — Source data [file 41467_2019_13960_MOESM7_ESM.zip › Source_Data/Figure6/Figure6B_MotifScanOutput/ETS1_PU.1/homerResults/motif2.similar.html]

motif2

## Information for motif2

C
T
A
G
T
G
A
C
G
T
C
A
C
T
A
G
T
C
A
G
G
T
C
A
C
G
A
T
T
A
C
G
A
G
C
T
A
C
T
G
A
C
T
G
A
C
G
T
A
G
C
T
C
G
A
T
  
Reverse Opposite:  

G
C
T
A
T
C
G
A
T
G
C
A
T
G
A
C
G
T
A
C
C
T
G
A
A
T
G
C
G
C
T
A
C
A
G
T
A
G
T
C
G
A
T
C
C
A
G
T
A
C
T
G
G
A
T
C
  

|  |  |
| --- | --- |
| p-value: | 1e-978 |
| log p-value: | -2.252e+03 |
| Information Content per bp: | 1.662 |
| Number of Target Sequences with motif | 5696.0 |
| Percentage of Target Sequences with motif | 35.98% |
| Number of Background Sequences with motif | 3186.9 |
| Percentage of Background Sequences with motif | 9.94% |
| Average Position of motif in Targets | 100.6 +/- 45.2bp |
| Average Position of motif in Background | 99.6 +/- 60.6bp |
| Strand Bias (log2 ratio + to - strand density) | 0.1 |
| Multiplicity (# of sites on avg that occur together) | 1.12 |
| Motif File: | file (matrix) reverse opposite |

### Similar de novo motifs found

|  |  |  |  |  |  |  |  |
| --- | --- | --- | --- | --- | --- | --- | --- |
| Rank | Match Score | Redundant Motif | P-value | log P-value | % of Targets | % of Background | Motif file |
| 1 | 0.794 | G C A T C G A T A C T G G C A T C T A G A C T G A G C T G C A T C G A T | 1e-880 | -2026.978734 | 38.50% | 12.61% | motif file (matrix) |
| 2 | 0.841 | C A G T C G A T C A T G A C T G G A T C C G A T A C T G A C G T A C T G A C T G A G C T G A C T C G A T | 1e-850 | -1958.544169 | 40.16% | 14.11% | motif file (matrix) |
| 3 | 0.773 | G C A T C T G A T C G A A G T C A G T C C G T A A G T C G C T A | 1e-845 | -1946.641984 | 40.78% | 14.60% | motif file (matrix) |
| 4 | 0.781 | T C G A T A C G G C T A C G T A T G C A A T G C G A T C C G T A A G T C C T G A | 1e-831 | -1913.471872 | 41.88% | 15.58% | motif file (matrix) |
| 5 | 0.771 | T G A C G T C A T A G C G C T A C T G A T C G A T G A C A G T C C T G A T A G C G C T A | 1e-816 | -1880.654528 | 41.72% | 15.65% | motif file (matrix) |
| 6 | 0.778 | C G A T A T C G G C A T A C T G A C T G A G C T A G C T C A G T A G T C A G C T A C G T C A G T | 1e-698 | -1607.554337 | 26.18% | 6.90% | motif file (matrix) |
| 7 | 0.764 | A C G T A C T G A G C T A C T G A C T G A C G T G A C T | 1e-645 | -1485.557372 | 27.64% | 8.32% | motif file (matrix) |
| 8 | 0.763 | C T G A T G C A A G T C A G T C C G T A T G A C G C T A | 1e-601 | -1385.282348 | 41.23% | 18.42% | motif file (matrix) |
| 9 | 0.817 | C G A T C A G T C A G T A T C G G C T A C G A T A T C G A C G T A C T G C T A G A C G T G C T A | 1e-573 | -1320.284063 | 32.94% | 12.72% | motif file (matrix) |
| 10 | 0.788 | A T C G A G T C T G A C C T G A A G T C C G T A A C G T A G T C A G T C A C T G A T C G A G T C A T G C | 1e-418 | -963.022102 | 15.86% | 3.96% | motif file (matrix) |
